# Supplementary figures and images for: Novel cell types and developmental lineages revealed by single-cell RNA-seq analysis of the mouse crista ampullaris
Source: eLife. 2021 May 18;10:e60108. doi: 10.7554/eLife.60108 (PMC8189719; doi:10.7554/eLife.60108)

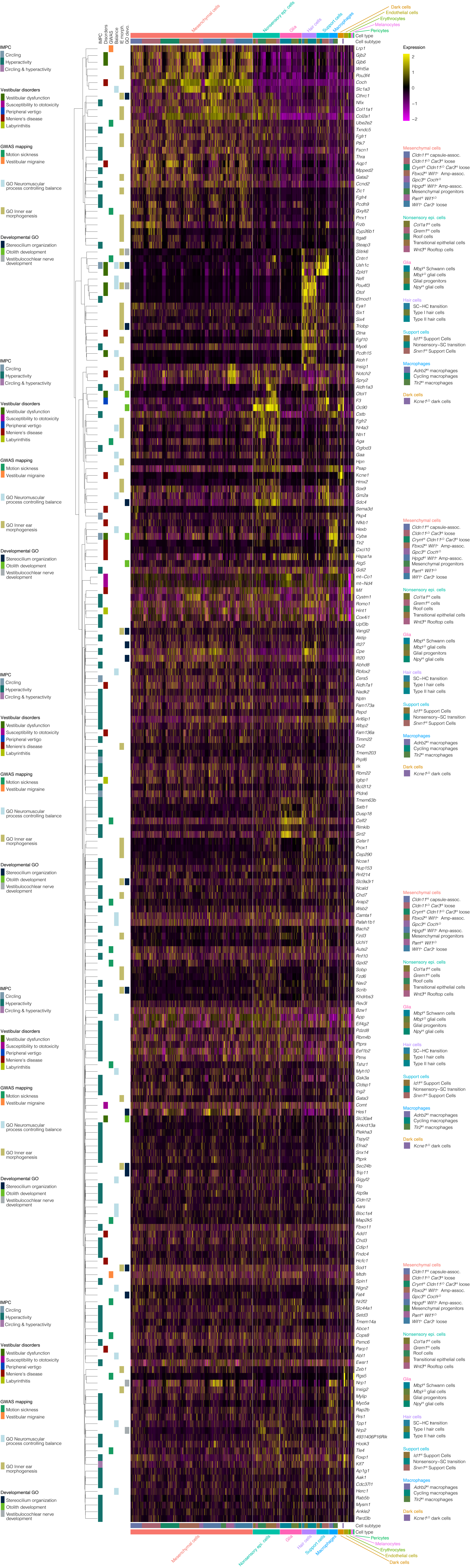

Supplement: Figure 9—source data 1. — The heatmap summarizes the expression of genes associated with vestibular disease and dysfunction in cell clusters of the crista ampullaris. Column color bars indicate the major cell types and subtypes. Row color bars denote the following: IMPC, genes mutated in mice that exhibit circling (cornflour blue) and hyperactivity (teal) or both (purple) phenotypes. Disorders, gene associations with Vestibular dysfunction (forest green), susceptibility to ototoxicity (magenta), peripheral vertigo (blue) and Meniere’s disease (maroon) were compiled from MSigDB, OMIM and Malacards databases. GWAS, hits in a genome-wide association studies of motion sickness (green) and vestibular migraine (orange). Balance, genes in the GO term ‘neuromuscular process controlling balance’ (sky blue). IE morph, genes in the GO term ‘inner ear morphogenesis’ (khaki). Devo., genes in GO terms ‘stereocilium organization’ (midnight blue), ‘otolith development’ (lime green) and ‘vestibulocochlear nerve development’ (gray). [file elife-60108-fig9-data1.pdf]
