# Supplementary material for: Novel cell types and developmental lineages revealed by single-cell RNA-seq analysis of the mouse crista ampullaris
Source: eLife. 2021 May 18;10:e60108. doi: 10.7554/eLife.60108 (PMC8189719; doi:10.7554/eLife.60108)
Supplement: Supplementary file 1. [file elife-60108-supp1.docx]

| **Supplemental file 1. Cell numbers, sequencing depth and gene detection in the crista ampullaris dataset.** | | | | | | | | | | | | | | | |  |
| --- | --- | --- | --- | --- | --- | --- | --- | --- | --- | --- | --- | --- | --- | --- | --- | --- |
|  | E16 | | |  | E18 | | |  | P3 | | |  | P7 | | | |
| *Cell type* | *Cell no.* | *Median counts per cell* | *Median genes per cell* |  | *Cell no.* | *Median counts per cell* | *Median genes per cell* |  | *Cell no.* | *Median counts per cell* | *Median genes per cell* |  | *Cell no.* | *Median counts per cell* | *Median genes per cell* | |
| *Adrb2*^HI^ macrophages | 4 | 1788 | 799 |  | 8 | 2924 | 1327 |  | 144 | 3527 | 1529 |  | 14 | 1627 | 882 | |
| *Cldn11*^HI^ capsule-assoc. mes. | 42 | 940 | 560 |  | 55 | 2287 | 1077 |  | 249 | 3415 | 1478 |  | 193 | 2260 | 1106 | |
| *Cldn11*^LO^ *Car3*^HI^ loose mes. | 105 | 736 | 442 |  | 91 | 2504 | 1084 |  | 336 | 3716 | 1507 |  | 236 | 1914 | 960 | |
| *Col1a1*^HI^ nonsensory epi. cells | 55 | 2265 | 1139 |  | 3 | 4018 | 1592 |  | 26 | 3943 | 1597 |  | 10 | 2091 | 1061 | |
| *Crym*^HI^ *Cldn11*^LO^ *Car3*^HI^ loose mes. | 145 | 697 | 439 |  | 72 | 2526 | 1199 |  | 368 | 3689 | 1536 |  | 183 | 1791 | 937 | |
| Cycling macrophages | 0 | NA | NA |  | 0 | NA | NA |  | 16 | 4673 | 1970 |  | 5 | 2566 | 1092 | |
| Dark cells | 85 | 1665 | 921 |  | 13 | 8723 | 2969 |  | 25 | 5295 | 2066 |  | 68 | 2248 | 1125 | |
| Endothelial cells | 75 | 986 | 526 |  | 12 | 3133 | 1429 |  | 37 | 6435 | 2494 |  | 66 | 2735 | 1254 | |
| Erythrocytes | 9 | 585 | 39 |  | 65 | 2219 | 61 |  | 26 | 2717 | 92 |  | 7 | 1804 | 811 | |
| *Fbxo2*^HI^ *Wif1*^+^ ampulla-assoc. mes. | 100 | 871 | 506 |  | 115 | 3366 | 1468 |  | 276 | 4416 | 1821 |  | 329 | 1887 | 954 | |
| Glial progenitors | 6 | 860 | 544 |  | 15 | 2499 | 1250 |  | 66 | 3228 | 1570 |  | 6 | 1827 | 978 | |
| *Gpc3*^HI^ *Coch*^LO^ capsule-assoc. mes. | 21 | 794 | 481 |  | 5 | 2767 | 1221 |  | 60 | 4050 | 1657 |  | 83 | 1714 | 937 | |
| *Grem1*^HI^ nonsensory epi. cells | 40 | 1612 | 843 |  | 14 | 7356 | 2521 |  | 19 | 4479 | 1792 |  | 29 | 5123 | 2064 | |
| *Hpgd*^HI^ *Wif1*^+^ ampulla-assoc. mes. | 149 | 912 | 534 |  | 82 | 2781 | 1317 |  | 325 | 4283 | 1743 |  | 178 | 2015 | 959 | |
| *Id1*^HI^ support cells | 73 | 1085 | 639 |  | 19 | 1963 | 1098 |  | 26 | 3542 | 1508 |  | 25 | 2580 | 1150 | |
| *Kcne1*^LO^ dark cells | 43 | 1001 | 584 |  | 5 | 3026 | 1201 |  | 2 | 24043 | 5100 |  | 3 | 3905 | 1747 | |
| *Mbp*^HI^ Schwann cells | 44 | 894 | 540 |  | 7 | 2272 | 1168 |  | 217 | 3323 | 1433 |  | 204 | 1950 | 999 | |
| *Mbp*^LO^ glial cells | 37 | 742 | 483 |  | 13 | 2086 | 1077 |  | 159 | 2166 | 1101 |  | 23 | 1600 | 897 | |
| Melanocytes | 6 | 1022 | 582 |  | 4 | 3220 | 1417 |  | 23 | 4454 | 1725 |  | 55 | 1843 | 880 | |
| Mesenchymal progenitors | 120 | 1402 | 747 |  | 35 | 4073 | 1685 |  | 36 | 6308 | 2428 |  | 16 | 2706 | 1269 | |
| Nonsensory–support cell transition | 86 | 938 | 560 |  | 0 | NA | NA |  | 1 | 4242 | 1749 |  | 2 | 2005 | 1010 | |
| *Npy*^HI^ glial cells | 8 | 695 | 431 |  | 16 | 1562 | 926 |  | 70 | 2070 | 1053 |  | 9 | 1514 | 796 | |
| *Pam*^HI^ *Wif1*^LO^ mesenchymal cells | 69 | 785 | 478 |  | 37 | 2511 | 1147 |  | 162 | 3361 | 1471 |  | 50 | 2112 | 1014 | |
| Pericytes | 14 | 787 | 480 |  | 12 | 2143 | 1071 |  | 14 | 2506 | 1221 |  | 7 | 1569 | 829 | |
| Roof cells | 58 | 1262 | 733 |  | 50 | 3022 | 1428 |  | 87 | 3814 | 1618 |  | 127 | 4338 | 1829 | |
| Support cell–hair cell transition | 79 | 6444 | 2570 |  | 9 | 17215 | 4606 |  | 10 | 13303 | 4064 |  | 11 | 7427 | 3150 | |
| *Srxn1*^HI^ support cells | 154 | 1573 | 888 |  | 20 | 4035 | 1682 |  | 56 | 3357 | 1357 |  | 58 | 3238 | 1477 | |
| *Tlr2*^HI^ macrophages | 16 | 800 | 452 |  | 14 | 2230 | 1047 |  | 129 | 3053 | 1263 |  | 12 | 1697 | 808 | |
| Transitional epithelial cells | 178 | 2157 | 1050 |  | 31 | 3777 | 1572 |  | 69 | 4886 | 1677 |  | 148 | 3151 | 1320 | |
| Type I hair cells | 92 | 1964 | 1047 |  | 5 | 4110 | 2054 |  | 20 | 3416 | 1637 |  | 30 | 2005 | 1184 | |
| Type II hair cells | 241 | 1207 | 717 |  | 12 | 3693 | 1656 |  | 49 | 3485 | 1608 |  | 46 | 2286 | 1284 | |
| Unidentified P7 cluster | 0 | NA | NA |  | 0 | NA | NA |  | 1 | 2883 | 1206 |  | 20 | 1850 | 1086 | |
| *Wif1*^+^ *Car3*^+^ loose mes. | 152 | 730 | 442 |  | 68 | 2731 | 1191 |  | 261 | 4054 | 1624 |  | 228 | 2054 | 1006 | |
| *Wnt3*^HI^ rooftop cells | 14 | 912 | 520 |  | 25 | 3819 | 1674 |  | 44 | 3989 | 1646 |  | 66 | 3169 | 1404 | |
