# Supplementary material for: Novel cell types and developmental lineages revealed by single-cell RNA-seq analysis of the mouse crista ampullaris
Source: eLife. 2021 May 18;10:e60108. doi: 10.7554/eLife.60108 (PMC8189719; doi:10.7554/eLife.60108)
Supplement: Supplementary file 2. [file elife-60108-supp2.docx]

| **Supplemental file 2. RNA ISH images from the Allen Developing Mouse Brain Atlas** | | | | | |
| --- | --- | --- | --- | --- | --- |
| Figure | Target | Stage | Image # | URL | Access date |
| Fig. 5S1 | *Alcam* | E15.5 | 1 | https://developingmouse.brain-map.org/experiment/show/100082551 | 2/4/21 |
| Fig. 4S1 | *Aldh1a1* | E15.5 | 3 | https://developingmouse.brain-map.org/experiment/show/100072741 | 2/4/21 |
| Fig. 4S1 | *Aldh1a3* | E15.5 | 3 | https://developingmouse.brain-map.org/experiment/show/100072748 | 1/10/20 |
| Fig. 2S2 | *Anxa4* | E15.5 | 1 | https://developingmouse.brain-map.org/experiment/show/100082537 | 3/28/20 |
| Fig. 5S1 | *Apod* | E15.5 | 1 | https://developingmouse.brain-map.org/experiment/show/100082645 | 1/10/20 |
| Fig. 2S2 | *Atoh1* | E15.5 | 3 | https://developingmouse.brain-map.org/experiment/show/100055556 | 3/28/20 |
| Fig. 7S1 | *C1qa* | E15.5 | 1 | https://developingmouse.brain-map.org/experiment/show/100082541 | 2/9/21 |
| Fig. 5S1 | *Car3* | E15.5 | 1 | https://developingmouse.brain-map.org/experiment/show/100072650 | 1/10/20 |
| Fig. 5S1 | *Cldn11* | E15.5 | 1 | https://developingmouse.brain-map.org/experiment/show/100085135 | 1/10/20 |
| Fig. 5S1 | *Coch* | E15.5 | 2 | https://developingmouse.brain-map.org/experiment/show/100083836 | 1/10/20 |
| Fig. 5S1 | *Col14a1* | E15.5 | 2 | https://developingmouse.brain-map.org/experiment/show/100057415 | 2/4/21 |
| Fig. 4S1 | *Col1a1* | E15.5 | 2 | https://developingmouse.brain-map.org/experiment/show/100058567 | 2/8/21 |
| Fig. 4S1 | *Col2a1* | E15.5 | 3 | https://developingmouse.brain-map.org/experiment/show/100057588 | 1/10/20 |
| Fig. 5S1 | *Col3a1* | E15.5 | 1 | https://developingmouse.brain-map.org/experiment/show/100057484 | 1/10/20 |
| Fig. 4S1 | *Col9a1* | E15.5 | 3 | https://developingmouse.brain-map.org/experiment/show/100037912 | 1/10/20 |
| Fig. 5S1 | *Col9a1* | E15.5 | 8 | https://developingmouse.brain-map.org/experiment/show/100037912 | 2/9/21 |
| Fig. 4S1 | *Col9a3* | E15.5 | 1 | https://developingmouse.brain-map.org/experiment/show/100083521 | 1/10/20 |
| Fig. 5S1 | *Col9a3* | E15.5 | 5 | https://developingmouse.brain-map.org/experiment/show/100083521 | 2/9/21 |
| Fig. 4S1 | *Crtac1* | E15.5 | 7 | https://developingmouse.brain-map.org/experiment/show/100037914 | 1/10/20 |
| Fig. 5S1 | *Crym* | E15.5 | 2 | https://developingmouse.brain-map.org/experiment/show/100085172 | 2/4/21 |
| Fig. 5S1 | *Dcn* | E15.5 | 2 | https://developingmouse.brain-map.org/experiment/show/100083827 | 1/10/20 |
| Fig. 3S1 | *Ednrb* | E15.5 | 3 | https://developingmouse.brain-map.org/experiment/show/100093067 | 2/4/21 |
| Fig. 7S1 | *Egr1* | E15.5 | 4 | https://developingmouse.brain-map.org/experiment/show/100085154 | 2/9/21 |
| Fig. 2S2 | *Ephb1* | E15.5 | 3 | https://developingmouse.brain-map.org/experiment/show/100053200 | 3/28/20 |
| Fig. 4S1 | *Gata2* | E15.5 | 2 | https://developingmouse.brain-map.org/experiment/show/100085333 | 1/10/20 |
| Fig. 5S1 | *Gpc3* | E15.5 | 5 | https://developingmouse.brain-map.org/experiment/show/100037929 | 1/10/20 |
| Fig. 2S2 | *Id1* | E15.5 | 1 | https://developingmouse.brain-map.org/experiment/show/100085141 | 3/28/20 |
| Fig. 4S1 | *Igfbp2* | E15.5 | 2 | https://developingmouse.brain-map.org/experiment/show/100085268 | 1/10/20 |
| Fig. 5S1 | *Igfbp2* | E15.5 | 2 | https://developingmouse.brain-map.org/experiment/show/100085268 | 2/4/21 |
| Fig. 2S2 | *Igfbp3* | E15.5 | 4 | https://developingmouse.brain-map.org/experiment/show/100047621 | 3/28/20 |
| Fig. 4S1 | *Igfbp4* | E15.5 | 5 | https://developingmouse.brain-map.org/experiment/show/100046957 | 1/10/20 |
| Fig. 2S2 | *Isl1* | E16.5 | 30 | https://developingmouse.brain-map.org/experiment/show/100091723 | 3/28/20 |
| Fig. 4S1 | *Mest* | E15.5 | 1 | https://developingmouse.brain-map.org/experiment/show/100072689 | 1/10/20 |
| Fig. 3S1 | *Npy* | E15.5 | 1 | https://developingmouse.brain-map.org/experiment/show/100084092 | 2/4/21 |
| Fig. 4S2 | *Nr4a3* | E11.5 | 1 | https://developingmouse.brain-map.org/experiment/show/100056709 | 4/20/20 |
| Fig. 4S1 | *Ntn1* | E15.5 | 1 | https://developingmouse.brain-map.org/experiment/show/100084124 | 1/10/20 |
| Fig. 4S2 | *Ntn1* | E11.5 | 2 | https://developingmouse.brain-map.org/experiment/show/100046576 | 4/20/20 |
| Fig. 3S1 | *Plp1* | E15.5 | 2 | https://developingmouse.brain-map.org/experiment/show/100098091 | 2/9/21 |
| Fig. 5S1 | *Pou3f4* | E15.5 | 2 | https://developingmouse.brain-map.org/experiment/show/100058667 | 1/10/20 |
| Fig. 2S2 | *Pou4f3* | E15.5 | 2 | https://developingmouse.brain-map.org/experiment/show/100057571 | 2/4/21 |
| Fig. 2S2 | *Rcn1* | E15.5 | 1 | https://developingmouse.brain-map.org/experiment/show/100047042 | 3/28/20 |
| Fig. 4S1 | *Rspo3* | E17.5 | 36 | https://developingmouse.brain-map.org/experiment/show/100091685 | 1/10/20 |
| Fig. 5S1 | *Sema3d* | E15.5 | 1 | https://developingmouse.brain-map.org/experiment/show/100047623 | 1/10/20 |
| Fig. 4S1 | *Sema3d* | E15.5 | 3 | https://developingmouse.brain-map.org/experiment/show/100047623 | 2/8/21 |
| Fig. 4S1 | *Sfrp5* | E15.5 | 1 | https://developingmouse.brain-map.org/experiment/show/100046053 | 1/10/20 |
| Fig. 5S1 | *Slc1a3* | E15.5 | 1 | https://developingmouse.brain-map.org/experiment/show/100083300 | 1/10/20 |
| Fig. 4S1 | *Smoc2* | E15.5 | 3 | https://developingmouse.brain-map.org/experiment/show/100045921 | 1/10/20 |
| Fig. 4S2 | *Smoc2* | E11.5 | 2 | https://developingmouse.brain-map.org/experiment/show/100046156 | 4/20/20 |
| Fig. 4S2 | *Sox2* | E11.5 | 2 | https://developingmouse.brain-map.org/experiment/show/100047263 | 4/20/20 |
| **Supplemental file 2. RNA ISH images from the Allen Developing Mouse Brain Atlas (*continued*)** | | | | | |
| Figure | Target | Stage | Image # | URL | Access date |
| Fig. 4S1 | *Spint2* | E15.5 | 2 | https://developingmouse.brain-map.org/experiment/show/100047217 | 1/10/20 |
| Fig. 4S1 | *Spp1* | E15.5 | 6 | https://developingmouse.brain-map.org/experiment/show/100084126 | 1/10/20 |
| Fig. 5S1 | *Tbx18* | E15.5 | 2 | https://developingmouse.brain-map.org/experiment/show/100055755 | 1/10/20 |
| Fig. 5S1 | *Wif1* | E15.5 | 1 | https://developingmouse.brain-map.org/experiment/show/100085187 | 1/10/20 |
| Fig. 4S1 | *Wnt3* | E15.5 | 2 | https://developingmouse.brain-map.org/experiment/show/100047803 | 1/10/20 |
| Fig. 4S2 | *Wnt3* | E11.5 | 1 | https://developingmouse.brain-map.org/experiment/show/100047117 | 4/20/20 |
